# *Legionella pneumophila* as a Health Hazard to Miners: A Pilot Study of Water Quality and QMRA

**DOI:** 10.3390/w11081528

**Published:** 2019-07-24

**Authors:** Valerie Madera-García, Alexis L. Mraz, Nicolás López-Gálvez, Mark H. Weir, James Werner, Paloma I. Beamer, Marc P. Verhougstraete

**Affiliations:** 1 Department of Community, Environment, and Policy, Mel and Enid Zuckerman College of Public Health, The University of Arizona, Tucson, AZ 85724, USA; 2 Department of Epidemiology and Biostatistics, College of Public Health, Temple University, Philadelphia, PA 19122, USA; 3 Division of Environmental Health Sciences, College of Public Health, The Ohio State University, Columbus, OH 43210, USA

**Keywords:** *Legionella pneumophila*, QMRA, mining safety, DALYs (disability adjusted life years), air quality ventilation

## Abstract

*Legionella pneumophila* (*L. pneumophila*), the causative agent of legionellosis, is an aquatic bacterium that grows in warm water. Humans are only presented with a health risk when aerosolized water containing *L. pneumophila* is inhaled. In mining operations, aerosolized water is used as dust control and as part of the drilling operations, a currently ignored exposure route. This study characterized *L. pneumophila* concentrations in the mine’s non-potable water and the relationship between *L. pneumophila* and chlorine concentrations. These concentrations informed a quantitative microbial risk assessment (QMRA) model to estimate the infection risk to miners exposed to aerosolized water containing *L. pneumophila*. Fourteen water samples were collected from seven locations at a mine and analyzed for temperature, pH, chlorine, and *L. pneumophila* serogroup. Most samples (93%) tested positive for *L. pneumophila* cells. The faucet from the sprinkler system on the adit level (entrance to the underground mine levels) showed the highest concentration of *L. pneumophila* (8.35 × 10^4^ MPN/L). Disability adjusted life years (DALYs) were estimated in the QMRA model and showed that the risk for all miners was significantly lower (*p* < 0.0001) with the ventilation system on than when the system was off. Our study showed that the use of a ventilation system at the adit level of the mine reduced the risk of infection with aerosolized *L. pneumophila*.

## 1. Introduction

*Legionella pneumophila* (*L. pneumophila*), a gram-negative, aquatic facultative bacterium is an intracellular parasite which multiplies in 13 species of amoeba commonly found in water-associated biofilms [1]. The bacterium thrives in large-premise plumbing systems as it colonizes biofilms and grows in warm water with temperatures between 25 and 45 °C [2–4]. Previous studies have reported the colonization of large water systems (e.g., hospitals and hotels) where *L. pneumophila* is dispersed via showerheads and faucets [3,5–8]. To cause illness, aerosolized *L. pneumophila* must be inhaled and deposited into the alveoli of the lung. Infection can result in legionellosis, a term which incorporates Legionnaires’ disease and Pontiac Fever [2,3,5,7,9]. *L. pneumophila* research has primarily focused on premise plumbing and cooling towers, which have shown an LD_50_ of approximately 100 organisms [10,11].

There are a variety of *Legionella* disinfection methods, with the most efficacious combining physical, chemical, and thermal methods [12]. Chemical methods, such as chlorination, must be effective in the elimination of water pathogens while leaving a residual level in the distribution system safe for human consumption. Chlorine is known to inactivate *L. pneumophila* in concentrations higher than those used in the domestic potable water (between 2 to 6 mg/L), and at temperatures above 41 °C [12]. Chlorine residuals dissipate throughout large-premise plumbing systems and decay faster at higher temperatures. Furthermore, chlorine residuals are shown to be less effective against bacteria residing in biofilms as the biofilm is impenetrable to the chlorine and the amoeba the bacteria utilize as host cells can be chlorine resistant [12]. There is limited knowledge regarding the persistence, distribution, and growth parameters of *L. pneumophila* in potable premise plumbing and water distribution systems. There is even less information on *Legionella* in non-potable water systems, such as misters and mines.

The extraction of minerals through mining activities is associated with the generation of dust, which is commonly controlled with aerosolized water [13]. Moreover, the quality of the water used in mining is often assessed based on environmental concerns, such as drainage into groundwater or the use of large open pits [14]. Concerns about the assessment of residual chlorine and water system maintenance in mine settings have not been addressed. A few studies have reported cases of Legionnaire’s disease among miners [15,16] and just one study tested water for *L. pneumophila* but failed to detect it [16]. Additionally, there are no published studies evaluating the risk of infection to *L. pneumophila* among mine workers using quantitative microbial risk assessment (QMRA).

QMRA is a computational science aimed at limiting infectious disease agents through an in-depth understanding of their biology and pathogenesis [17]. It has been proven effective for analytical and decision support in multiple exposure scenarios in various environmental medias, including air and water [18–20]. QMRA is a vital tool in understanding the risk in specific exposure scenarios and providing stakeholders with the information needed to make informed decisions on how to mitigate the potential for disease.

There is a lack of knowledge regarding the risk of legionellosis to miners. This study assessed the risk of exposure to *L. pneumophila* and the associated risk of a disability-adjusted life year (DALY) of miners exposed to aerosolized water using QMRA. A mine in southwest United States uses stored, non-chlorinated water for a dust-suppressing sprinkler system during mining activities in all working levels of the mine, resulting in the aerosolization of water. The non-potable water may remain in a non-covered storage tank for up to six months before being used or replaced. These practices can promote the growth of biofilms and *L. pneumophila*. This study characterized *L. pneumophila* concentrations in non-potable water used in the mine and the relationship between these *L. pneumophila* concentrations and chlorine in the water and then utilized a QMRA model to estimate the infection risk to miners exposed to aerosolized water containing *L. pneumophila*.

## 2. Methods

### 2.1. Site Description Section

This study was conducted at a 90-acre underground mine in the southwest region of the United States with four working levels and a depth of 250 feet. The facility has three water tanks: potable water, non-potable water, and non-potable water exclusively for firefighting. Each tank has a capacity of 22.7 m^3^, is refilled every 6 months, and none of the tanks receive any on-site chlorination treatment. A dust-suppressing sprinkler system drawing from the non-potable water tank was installed, but not utilized prior to the current study, resulting in stagnant water within the system. The jackleg drills at the studied mine utilized water from the non-potable water tank to abate dust and keep equipment clean, resulting in aerosolized water exposure to the surrounding workers. The non-potable water tank supplies the hoist house, adit level (first level on the interior of the mine), and underground levels. Seven sampling locations were chosen based on the water usage, storage conditions, potential exposure to aerosolized water, and proximity and accessibility to miners.

### 2.2. Sample Collection

**Figure 1.**
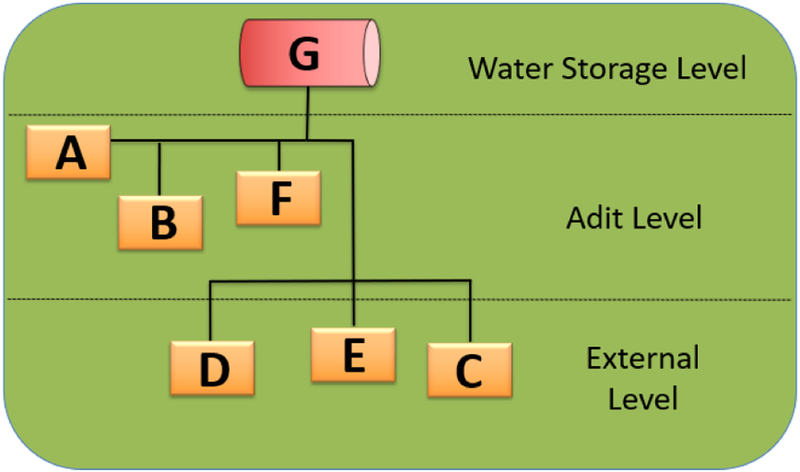
Diagram of water distribution system and sampling sites at the studied mine.

Each selected location was sampled in duplicate in March 2017. A total of 14 water samples were collected for the *L. pneumophila* culture analysis. Temperature and chlorine levels were recorded for each sampling location. Multiple pipes distribute the water downstream from the non-potable water tank (Figure 1), through the adit level (entrance of the underground mine levels) and the exterior level (open field including the hoist house). The sampling locations included: the (A) sprinkler system on the adit level, (B) jackleg drill connection in adit level, (C) swamp cooler, (D) sink at hoist house, (E) jackleg drill hose at hoist house, (F) jackleg drill hose outside adit level, and (G) non-potable water tank.

Sterile, one-liter sealable plastic bottles, containing 0.1 N sodium thiosulfate, were used to collect approximately 1 liter of water after flushing the system for 1 min [21]. Water samples were collected from the non-potable tank by submerging one-liter plastic bottles directly into the tank. An additional water sample from each location was collected in a separate sterile plastic bottle to measure chlorine levels on-site using a pool chlorine test kit and temperature using a handheld thermometer [21]. All one-liter samples were immediately placed inside a cooler with ice packs to prevent rising temperatures and the potential growth of *L. pneumophila* due to the warm climate of Arizona. All samples were transported to the University of Arizona’s Medical Research Building (Tucson, Arizona, USA), and processed upon arrival.

### 2.3. Laboratory Analysis

The Legiolert® test kit by IDEXX was used to quantify *L. pneumophila* (detects 1 organism in 100 mL within 7 days) in non-potable water following manufacturer recommended methods, briefly described here [22]. A volume of 100 mL of deionized water and the content of a Legiolert® blister pack were added to a specimen container. Using a sterile microtube, 0.2 mL of Legiolert® Pretreatment and 0.2 mL of non-potable water sample were mixed and incubated at room temperature for 60 s (±5 s). Approximately 0.2 mL of the mix contained in the microtube was transferred to the specimen container. After mixing, the contents of the specimen containers were poured into Quanti-Tray/Legiolert® trays. The trays were immediately sealed in an IDEXX Quanti-Tray Sealer PLUS (IDEXX Laboratories, Inc., Westbrook ME, USA), and incubated paper-side down with the wells facing up at 37 ± 0.5 °C, 85% humidity for 7 days. For comparison, a negative control blank containing Legiolert® reagent and deionized water was incubated. Any sample that exhibited a brown color or a turbidity higher than the negative control was positive for *L. pneumophila*. A lack of brown color change and turbidity less than the negative control indicated a negative result. All the positive wells were counted to obtain the concentration in most probable number (MPN) as indicated by the Legiolert® MPN table (dilution factor = 0.0001).

### 2.4. Data Analysis

Arithmetic averages were calculated to summarize the concentrations of *L. pneumophila*. The concentration results were grouped into three categories based on their location (water storage level, adit level, and exterior level) to determine which areas represent a higher risk of exposure to miners. All analyses were performed using R [23].

### 2.5. QMRA

The QMRA focused on the sprinkler and jackleg drill, as these two systems aerosolize water known to contain *L. pneumophila*, therefore posing the highest risk of legionellosis to miners [23]. This model evaluated the risk of infection based on the concentration of aerosolized *L. pneumophila* as well as the probability of illness and the DALYs based on the risk of infection. According to the mine’s manager, the sprinkler system should be activated for 10 min prior to workers entering the mine and workers used the jackleg drill for 4-hour shifts up to twice a week. The concentration of respirable (≤5 μm) *L. pneumophila* (MPN/L) released into the mine from the sprinkler system every second (*CL,air,S*) was calculated following Equation (1), where (*C*_*L,air,S*_)is the *L. pneumophila* concentration in the non-potable water feeding the sprinkler system (MPN/L), *PC* is the air partitioning coefficient (unitless), *V*_*S*_ is the volume of water released from the sprinkler (L/s), *F*_*A*_ is the fraction of aerosolized organisms 1–5 μm (unitless), and V_m,air_ is the area of the mine (L).

(1) $$C_{L,air,S}\;=\;\frac{C_{L,S}\cdot PC\cdot V_{s}\cdot F_{A}}{V_{m,air}}$$

The concentration of respirable *L. pneumophila* (MPN/L) released into the mine due to the jackleg drill (*C*_*L,air,JD*_) was calculated using Equation (2), where *C*_*L,JD*_ is the *L. pneumophila* concentration in the non-potable water feeding the jackleg drill (MPN/L), *PC* is the air partitioning coefficient (unitless), *V*_*JD*_ is the flow of water released from the jackleg drill (L/s), *F*_*A*_ is the fraction of aerosolized organisms 1–5 μm (unitless), and V*m,air* is the area of the mine (L).

(2) $$C_{L,air,JD}\;=\;\frac{C_{L,JD}\cdot PC\cdot V_{JD}\cdot F_{A}}{V_{m,air}}$$

The mine utilizes a ventilation fan which removes ~5600 liters of air per second. The amount of *L. pneumophila* vented (*N*_*vent*_) was calculated using Equation (3), where *V*_*v*_ is the volume of air the ventilation fan is removing from the mine (L) calculated using the fan flow rate over an 8-hour workday.

(3) $$N_{vent}\;=\;\left(C_{L,air,S}\;+\;C_{L,air,JD}\right)\cdot V_{v}$$

The *L. pneumophila* infectious dose to an individual (*d*) was calculated by summing the concentration of *L. pneumophila* in the mine per second the individual is assumed to be in the mine (one 4-hour shift) and multiplying the dose by the percentage of infectious *L. pneumophila* in a typical water system (10^−4^) and the inhalation rate utilizing Equation (4) [24]. Inhalation rates for male (*I*_*male*_) and female (*I*_*female*_) construction workers (m^3^/min) were used as construction was the most similar field to mine workers in the exposure factors handbook [25]. Equation (4) includes the concentration of respirable *L. pneumophila* into the mine from the sprinkler system during a shift, MPN/L (*C*_*L,air, S*_), the concentration of respirable *L. pneumophila* released into the mine due to the jackleg drill during a shift, MPN/L (*C*_*L,air, JD*_), the percent of *L. pneumophila* assumed infectious randomly selected from a uniform distribution of 0 to 1 (*I*), the inhalation rate, L/s (*R*_*I*_), and the amount of time a person is in the mine, seconds (*t*).

(4) $$d\;=\;\left[\left(C_{L,\text{air},S}\;+\;C_{L,air,JD}\right)\cdot I\cdot R_{I}\cdot t\right]\;-C_{vent}$$

The daily infectious risk (*P*_*i*_) was calculated using Equation (5), where *k* is the probability of the pathogen surviving to initiate infection in the host, and *d* is the inhaled dose calculated with Equation (4).

(5) $$P_{i}\;=\;1\;-\;{\text{e}}^{-k\cdot d}$$

The probability of illness given infection was modeled using a simulated morbidity ratio (*MR*) due to the lack of a known morbidity ratio for the study’s diverse population. This *MR* is simulated using a uniform distribution parameterized from 0 to 1. Equation (6) shows the product used to calculate the probability of illness given infection (*P*_*ill*_). This *P*_*ill*_ is then annualized (*P*_*ill,ann*_) using Equation (7) to calculate disability adjusted life years (DALYs) where *j* is progressed from 1 to the maximum number of exposure hours per year, assumed to be 400 h (4-h work shifts, 2 work shifts per week for 50 weeks per year, assuming 2 weeks o per year). DALYs (Equation (8)) were calculated utilizing the disability weights (DWs) presented in Table 1 for moderate infectious disease used to simulate Pontiac Fever (PF), severe infectious disease used to simulate Legionnaires’ disease (LD), and for post-acute consequences or sequela (e.g., long-term pulmonary damage).

(6) $$P_{ill}\;=\;P_{i}\cdot MR$$

(7) $$P_{ill,ann}\;=\;1-\prod_{1}^{j}\left(1-P_{ill}\right)$$

(8) $$DALY\;=\;P_{ill,am}\cdot DW$$

## 3. Results

### 3.1. Characterization of Legionella pneumophila Concentration

*L. pneumophila* was detected in 93% of the 14 water samples tested; one sample from the jackleg drill hose outside adit level was negative. Table 2 shows the results of temperature, chlorine levels, *L. pneumophila* average concentration at each sampling location, and the categorization of sampling locations based on the mine levels. The highest *L. pneumophila* average concentration, 8.35 × 10^4^ MPN/L, was found in the sprinkler system on the adit level of the mine, where chlorine levels were below detection limit. The lowest *L. pneumophila* average concentration was found in the non-potable water tank that supplies all the tested sampling locations (1.50 × 10^4^ MPN/L). The jackleg drill hose on the adit level of the mine had the highest tempreature amongs (30.9 °C)

**Figure 2.**
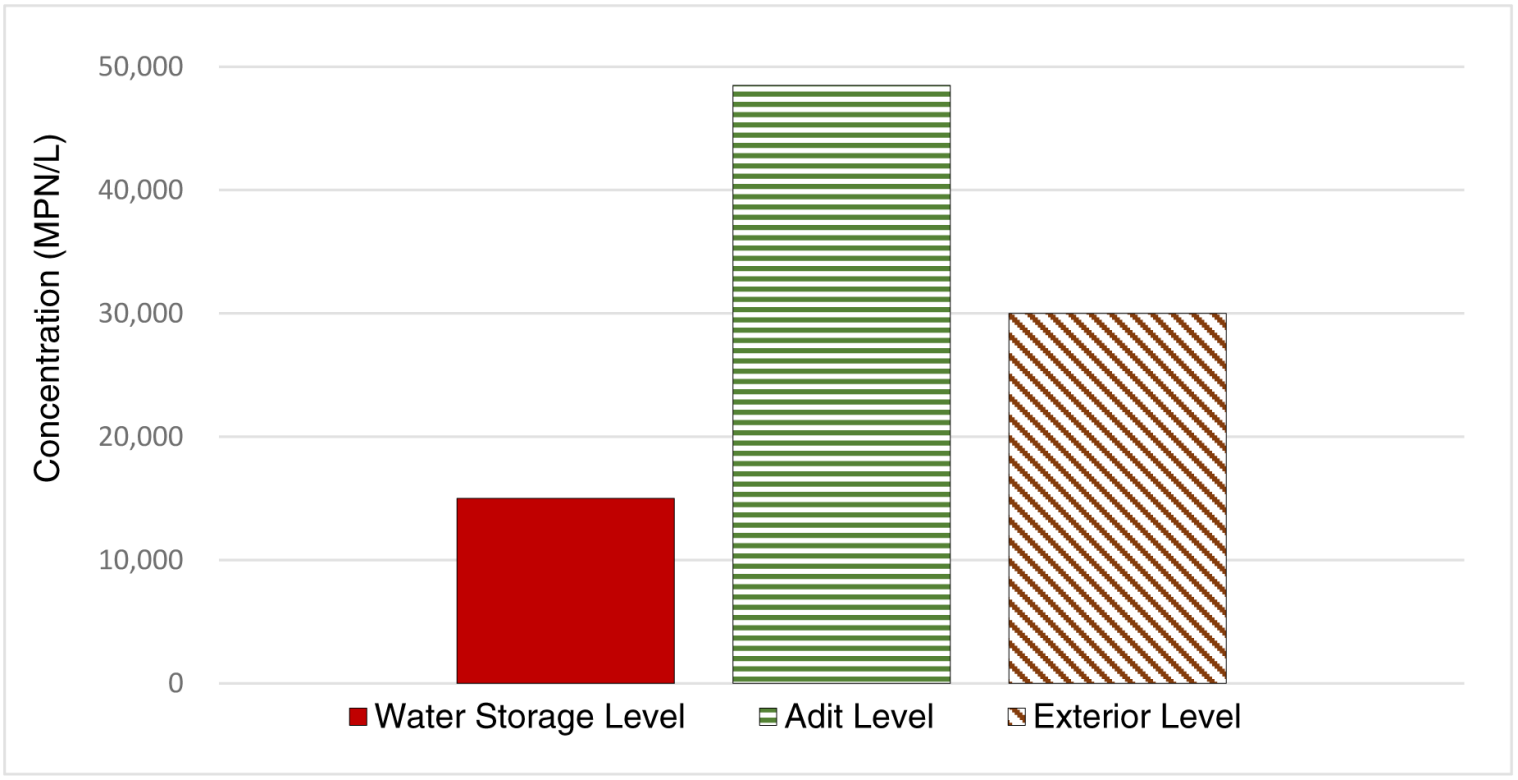
Average *L. pneumophila* concentrations on water storage level (n = 1), adit level (n = 3), and exterior level (n = 3).

After categorizing the sample locations based on the mine levels, the highest *L. pneumophila* average concentration (4.88 × 10^4^ MPN/L) occurred on the adit level, where water aerosolization occurs through jackleg drilling and dust control with the sprinkler system (Figure 2). Therefore, a QMRA model was built to estimate the risk of exposure to aerosolized *L. pneumophila* on the adit level of mine.

### 3.2. QMRA Model

**Figure 3.**
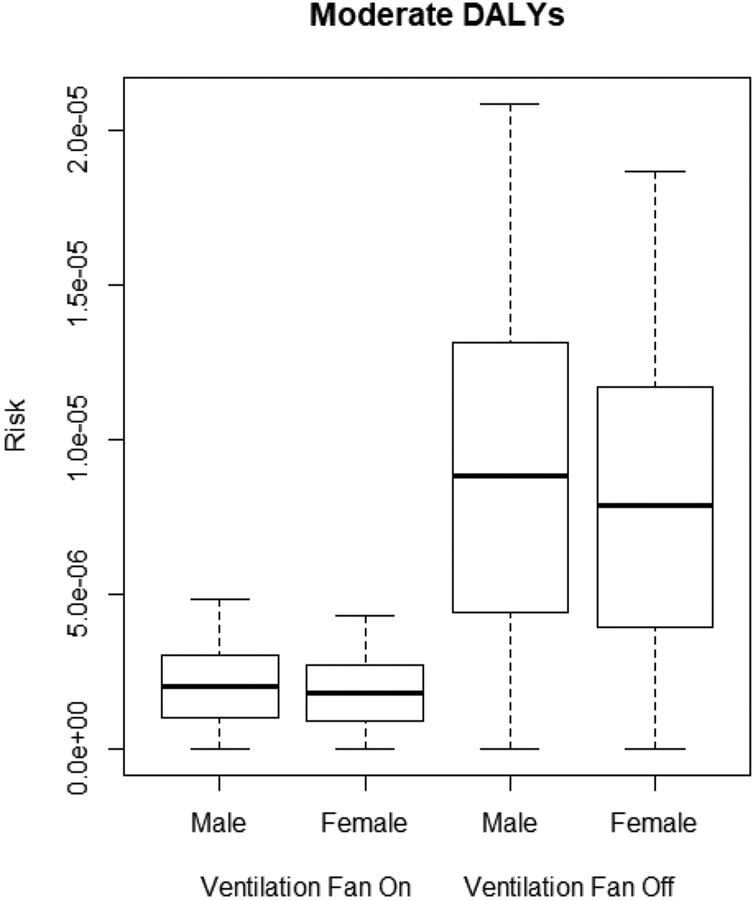
Moderate disability adjusted life years (DALYs) for male and female miners.

**Figure 4.**
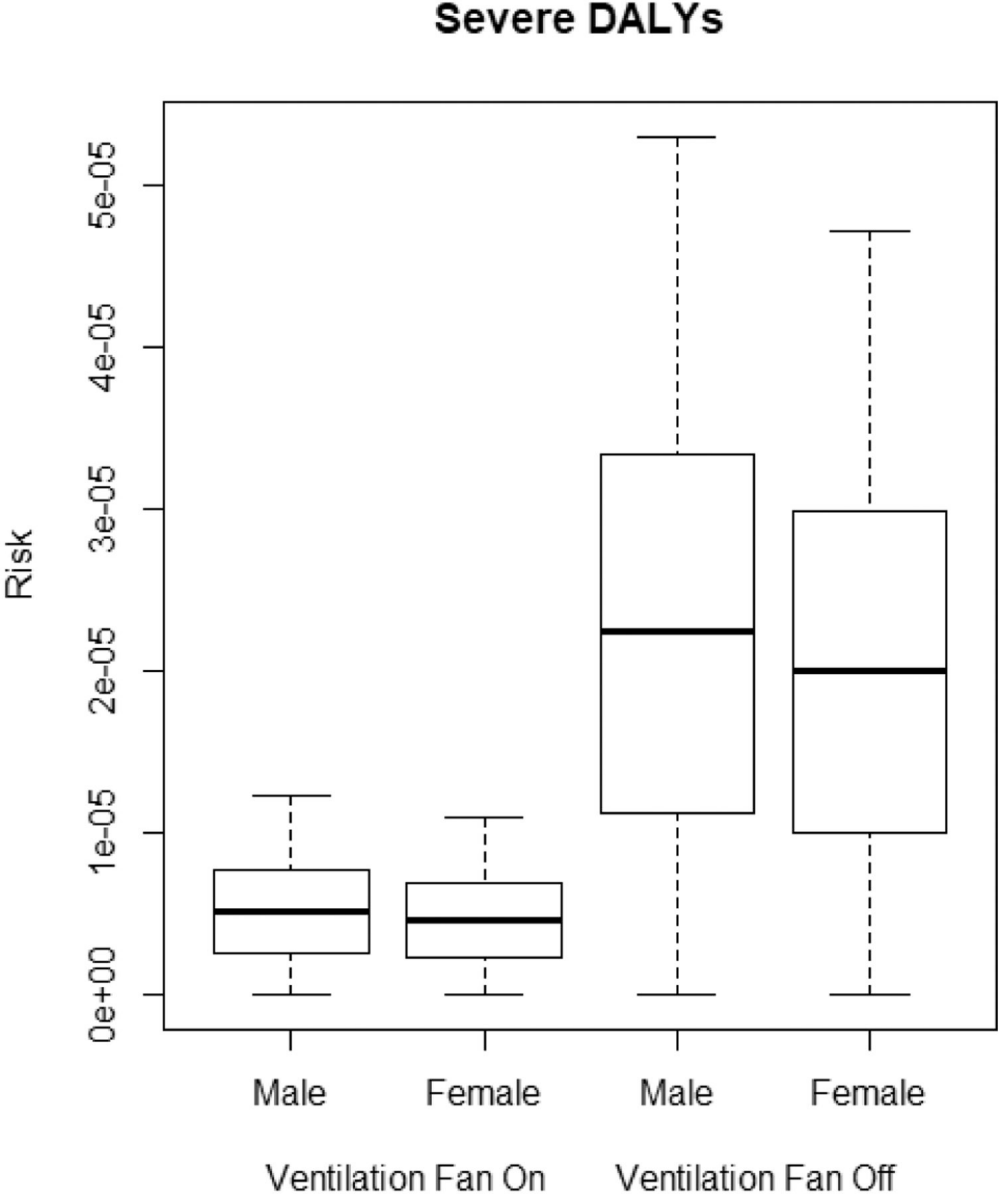
Severe DALYs for male and female miners.

**Figure 5.**
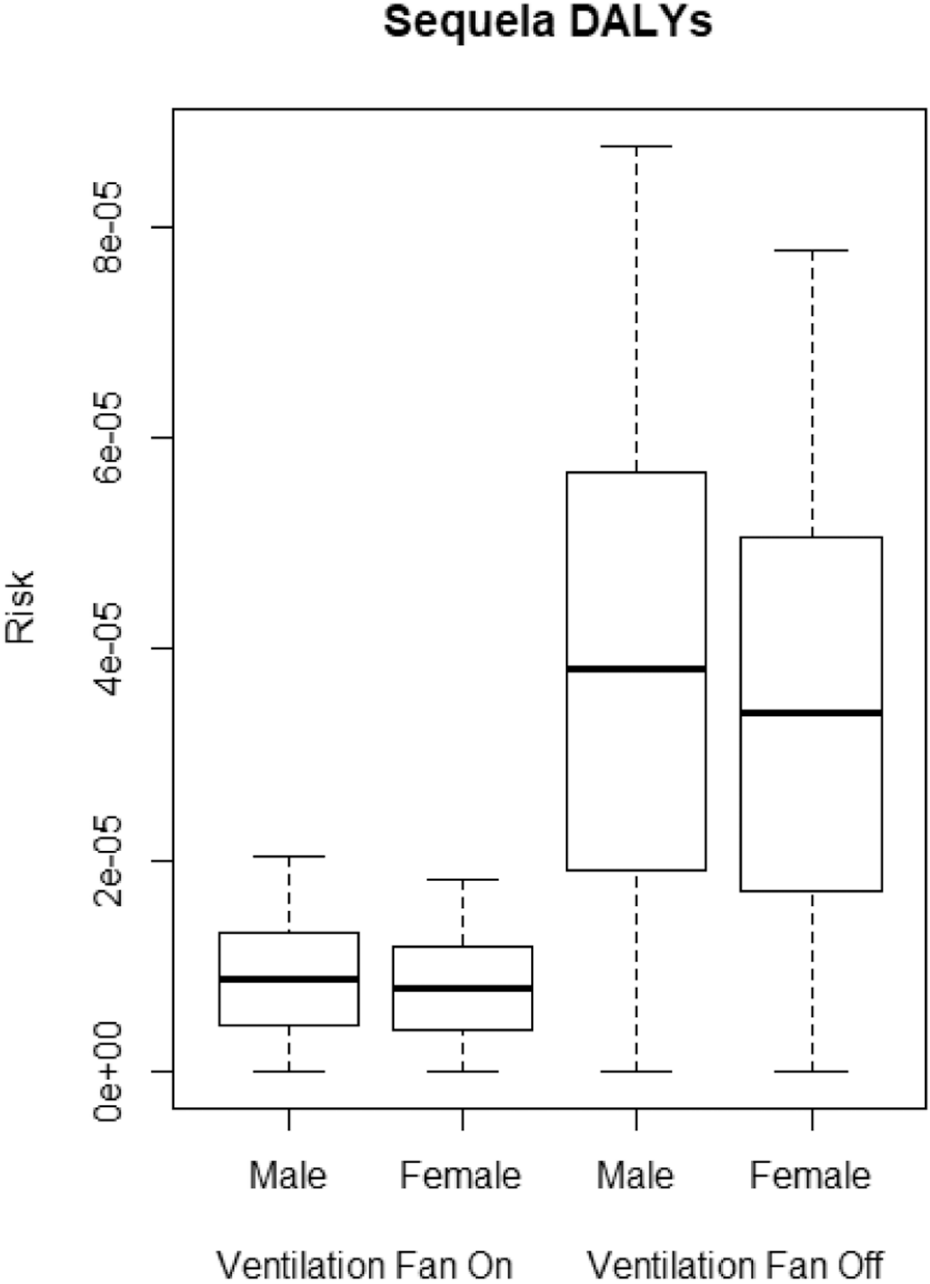
Sequela DALYs for male and female miners.

The QMRA model describes the DALYs of male and female miners due to legionellosis based on *L. pneumophila* concentrations in the sprinkler and jackleg drill connections shown in Table 2. Male miners are at higher risk of legionellosis than female miners, as seen in the moderate, severe, and sequela DALYs (*p* < 0.0001). The ventilation system was effective at significantly lowering the moderate, severe, and sequela DALYs for both male and female miners (*p* < 0.0001) as shown in Figures 3–5.

With the ventilation system off, DALYs ranged from 7.86 × 10^−6^ to 2.24 × 10^−5^. With the ventilation system on, DALYs were significantly lower (*p* < 0.001), with risk levels ranging from 1.81 × 10^−6^ to 8.79 × 10^−6^.

## 4. Discussion

This study characterized *L. pneumophila* concentrations in non-potable water used in the mine and estimated the infection risk to exposed miners using a QMRA model. *L. pneumophila* was detected in nearly all non-potable water samples. Furthermore, the highest average concentration of *L. pneumophila* was found in the sprinkler system on the adit level, which had not been previously used or flushed. *L. pneumophila* average concentrations were higher on the adit level compared to the other mine levels. Miners spend most of their work shift on this level and more water volume was observed to be used, which increases their risk of exposure to aerosolized *L. pneumophila*. There are no published reports which the authors are aware of that examines the presence of this bacterium in mine water.

A total of 86% of the locations sampled were found to have temperatures below the temperature range preferred for *L. pneumophila* growth (25–45 °C) [8]. Nevertheless, all tested locations were positive for the presence of *L. pneumophila*. The highest temperature, 30.9 °C, was measured at the jackleg drill hose outside the adit level which is consistently exposed to sun light. A typical dose of 0.1 to 10 mg/L of chlorine is effective and widely used against *Legionella* [12]. Approximately 71% of the sampling locations had chlorine residuals below detection limits, indicating a deficiency in chlorination of the non-potable water supply. One limitation of the study is that the Legiolert® test kit is designed to detect *L. pneumophila* but not to distinguish between serotypes. However, this is accounted for in the model by using the variable of the percentage of infectious *L. pneumophila* which includes all serotypes.

The QMRA shows male miners have higher DALYs due to legionellosis than female miners in all three categories (e.g., moderate, severe, and sequela), which corresponds to surveillance data showing men have higher incidences of legionellosis as compared to women [30]. In the QMRA, this result is directly linked to higher inhalation rates in males, leading to a higher potential dose of *L. pneumophila*. With or without the use of the ventilation system, male and female DALYs were above the acceptable risk of 1.0 × 10^−6^ in each category. DALYs results suggest that a lack of chlorine and the presence of *L. pneumophila* in non-potable water in the mine presents an unacceptable risk of legionellosis to miners. Considering non-potable water is used in the sprinkler system and jackleg drill, both of which produce aerosols, it is recommended that the water used for these activities be treated to avoid hazardous levels of *L. pneumophila* and reduce the risk of legionellosis to miners even if it is not intended for consumption.

Ventilation systems are often used to improve the quantity and quality of airflow throughout the underground mining levels by diluting and removing dangerous gases, reducing air temperature where mining machineries are being used, and providing airflow to unventilated areas [31,32]. In the current study, the mine’s ventilation system is only an exhaust fan. It does not represent a moisture control dynamic and should not be confused with HVAC (heating, ventilation, and air conditioning) systems, which some studies have demonstrated increase legionellosis risks in building settings [33,34]. Our study showed that the use of a ventilation system at the adit level of the mine reduces the risk of infection with aerosolized *L. pneumophila*. These results should be verified to be consistent for those mine ventilation systems which include moisture controls, that are more akin to HVAC systems.

Although the mechanism of the transmission of *L. pneumophila* can be di cult to identify, devices aerosolizing water have been involved in transmitting this bacterium to workers from a variety of occupational settings [35–37]. A literature review conducted on the cases of occupational legionellosis from 1978 to 2016 found that the workplaces most frequently associated with occupational legionellosis were industrial settings (62.0%), such as electrical power generation facilities, oil drilling, and automobile manufacturing, followed by office buildings (27.3%) and health care facilities (6.3%) [38]. Other workplaces, such as artesian excavation, horticultural sites, and sewage plants, reported sporadic cases of legionellosis [38]. The most prevalent sources of legionellosis infection in all workplaces were related to poorly maintained water sources such as coolant systems, cooling towers, spas, and vegetable misters [36–39]. To our knowledge, this is the first study that has detected *L. pneumophila* in a water system used in a mine setting. The findings of this study highlight the importance of maintaining and monitoring non-potable water sources that have the potential to produce aerosols. This study also highlights the risk of acquiring legionellosis in non-office environments. Even though there are no specific OSHA standards for the presence of *L. pneumophila* in occupational settings, this agency has a general duty clause of providing working environments free of known hazards related to the water sources and provide maintenance of all water systems to prevent growth of *L. pneumophila* [40].

Further research should focus on additional sampling throughout the mine, including the underground levels of the mine, where miners can spend part of their work shift. Non-potable water samples pre- and post-chlorine treatment should be assessed to determine the concentrations of chlorine necessary to prevent *L. pneumophila* from colonizing the distribution system.

## Figures and Tables

**Table 1.** Parameters used in the quantitative microbial risk assessment (QMRA) model.

Label	Variable	Value	Unit	Distribution	Reference
*C*_*L,S*_	Concentration of *L. pneutnophila* in non-potable water from the sprinkler system	8.35 × 10^−4^	Most probable number (MPN)/L	N/A	Results of this study
*PC*	Air partitioning coefficient	10^−5^	Unitless	N/A	[26]
*V*_*S*_	Water volume released from the sprinkler system	573	L/s	N/A	Provided by the mine
*F*_*A*_	Fraction of aerosolized organisms	Mean: 0.0337	Unitless	Normal	[27]
		Std: 0.0098			
*V*_*m,air*_	Area of the mine	3.0 × 10^−4^	L	N/A	Provided by the mine
*V*_*v*_	Volume of air removed by the ventilation fan	5663	L/s	N/A	Provided by the mine
*Cl,jd*	Concentration of *L. pneutnophila* in non-potable water from the jackleg drill	4.65 × 10^−4^	MPN/L	N/A	Results of this study
*V*_*JD*_	Flow of water released from the jackleg drill	573	L/s	N/A	Provided by the mine
*I*_*male*_	Inhalation rate for male workers	Mean: 0.02333	m^3^/min	Normal	[25]
		Std: 0.00434			
*I*_*female*_	Inhalation rate for female workers	Mean: 0.02083	m^3^/min	Normal	[25]
		Std: 0.0056			
*k*	Probability of the pathogen surviving to initiate infection in the host	−0.00599	Unitless	N/A	[28]
*DW*_*PF*_	Disability weight for Pontiac Fever (moderate)	Mean: 0.051	Unitless	Triangular	[29]
		Lower: 0.039			
		Upper: 0.06			
*DW*_*LD*_	Disability weight for Legionnaires′ disease (severe)	Mean: 0.125	Unitless	Triangular	[29]
		Lower: 0.104			
		Upper: 0.152			
*DW*_*PC*_	Disability weight for sequela (post-acute)	Mean: 0.217	Unitless	Triangular	[29]
		Lower: 0.179			
		Upper: 0.251			

**Table 2.** Water and quality measurements and the concentration *L. pneumophila* in non-portable water sources.

Sampling Location	Water temperature (°C)	Chlorine levels (mg/L)	Average Concentration of *L. pneutnophila* (MPN/L)	Categories of Sampling Location by Mine Levels
Non-potable water tank	23.6	ND	1.50 × 10^4^	Water Storage Level
Jackleg drill connection	21.6	ND	4.65 × 10^4^	Adit Level
Sprinkler system	21.1	ND	8.35 × 10^4^	Adit Level
Jackleg drill hose outside adit level	30.9	ND	1.55 × 10^4^	Adit Level
Sink in hoist house	19.0	0.05	2.50 × 10^4^	Exterior Level
Swamp cooler	16.8	0.05	4.00 × 10^4^	Exterior Level
Jackleg drill hose in hoist house	23.3	ND	2.50 × 10^4^	Exterior Level

ND: Not detected.
